# Factors influencing the decision to opt out of basic medical insurance among China’s migrant population: a logistic regression analysis

**DOI:** 10.3389/fpubh.2026.1787324

**Published:** 2026-03-24

**Authors:** Xiangyu Li, Haiwei Zuo, Mingchao Li

**Affiliations:** 1 School of Business, Shenzhen City Polytechnic, Shenzhen, Guangdong, China; 2 School of Information and Communications Technology, Shenzhen City Polytechnic, Shenzhen, Guangdong, China; 3 Office of Planning and Development, Shenzhen City Polytechnic, Shenzhen, Guangdong, China

**Keywords:** basic medical insurance, influencing factors, logistic regression model, migrant population, opting-out behavior

## Abstract

**Background:**

For countries with large internal migrant populations, reducing the opt-out rate from basic medical insurance among this group remains a major obstacle to achieving universal health coverage (UHC). Within China’s basic medical insurance system, migrants whose registered household locations differ from their places of residence have comparatively high opt-out rates. If they forgo their entitlement to basic medical insurance, they may face substantial out-of-pocket medical costs and the risk of being unable to access necessary healthcare services when suffering from serious illnesses. This study conceptualizes opting out as a multistage decision-making process influenced by rational and irrational factors. By identifying key associated factors of opting out among migrants, it aims to inform targeted policies to improve their insurance coverage.

**Methods:**

The analytical sample originates from the 2018 China Migrant Population Dynamic Monitoring Survey. After data cleaning and excluding respondents with unclear insurance status, 144,048 valid cases were identified, including 8,355 respondents who reported no enrollment in any scheme. This study first conducted a descriptive analysis of opt-out rates across different migrant groups, across demographic, migration, economic, employment, and health characteristics. Subsequently, a binary logistic regression model was employed to explore the key determinants of opting out. The final model included 11 explanatory variables (age group, educational attainment, marital status, migration duration, mobility range, reason for migration, expected local stay duration, household income, household expenditure, employment status, and recent health status), with the region of residence as a control variable.

**Results:**

Descriptive analysis revealed an overall opt-out rate of 5.80% among the migrant population. Opt-out rates varied across the demographic groups. Binary logistic regression analysis further indicated that the probability of opting out was significantly associated with multiple factors, including age, educational attainment, marital status, migration distance, income level, employment stability, and health status. For example, compared with migrants aged 15–19, those aged 60 or older had lower odds of opting out (OR = 0.403, *p* < 0.001). Compared with interprovincial migrants, intra-city cross-county migrants had lower odds (OR = 0.428, *p* < 0.001). Higher household income and employment were associated with lower odds (household income ≥20,000 yuan/month: OR = 0.470, *p* < 0.001; workers without a fixed employer vs. unemployed: OR = 0.736, *p* < 0.001). Additionally, migrants without recent illness (no recent illness vs. illness requiring hospitalization: OR = 1.320, *p* < 0.001) were more likely to opt out, consistent with some degree of adverse selection.

**Conclusion:**

Under China’s current basic medical insurance system, opt-out behavior among the migrant population is the result of the combined effects of the multidimensional individual variables. To effectively reduce the opt-out rate, targeted adjustments to the current medical insurance policies should be proactively made, thereby advancing the full achievement of UHC.

## 1 Introduction

Under China’s current basic medical insurance system, every member of society can enroll in a basic medical insurance program corresponding to their status. However, due to the lack of comprehensive mandatory enrollment requirements, some individuals have neither enrolled in employment-related employee medical insurance nor in the “annual payment” urban and rural residents’ medical insurance (1, 2). The decision to forgo enrollment in basic medical insurance constitutes opting out (3). It should be emphasized that the “opting-out behavior” referred to in this study primarily aims to explore individuals’ attitudes and choices towards basic medical insurance vigorously promoted by the government, without considering individuals’ participation in supplementary commercial medical insurance for the time being (4). For migrant populations, the disconnect between their actual residence and registered household location is associated with significant challenges in enrollment and limits access to insurance benefits, and is correlated with a higher opt-out rate compared to the general population (5, 6).

Once migrants opt out, they effectively exit the government-led, public-interest-oriented medical security system. Should they encounter serious illness, they may be unable to afford the necessary medical treatment, increasing the risk of medical impoverishment (7, 8). To prevent this phenomenon, the Chinese government has promoted the Universal Coverage Plan, striving to increase health insurance enrollment rates among the migrant population (9). This objective aligns with the core requirement of Sustainable Development Goal 3.8 (SDG 3.8)—Universal Health Coverage (UHC).

Regarding opting out of basic medical insurance, the academic discourse currently centers on two primary interpretive frameworks. Early perspectives viewed opting out as an adverse selection outcome stemming from individual rational analysis. Adverse selection theory, proposed by Akerlof (10) through his analysis of substandard markets under asymmetric information, was subsequently applied to health insurance research. Scholars have examined whether individuals with different health statuses, when possessing informational advantages, would make enrollment choices detrimental to market efficiency. Most studies concluded that under conditions of incomplete information, high-risk groups are more inclined to purchase insurance, forcing insurers to raise premiums and ultimately driving low-risk groups out of the insurance market (11, 12). In China, where basic medical insurance offers significant voluntary choice, individual health status has been found to significantly influence enrollment decisions (4).

As the rational agent assumption waned, prospect theory emerged as another crucial analytical tool for examining opting-out behavior. In 1979, Kahneman and Tversky (13) proposed prospect theory to explain irrational behavior by revising traditional assumptions about market agents’ rationality, self-interest, utility maximization, and preference consistency. Numerous studies have applied this theory’s concepts of probabilistic subjective weighting and loss aversion to elucidate the mechanisms underlying health insurance selection inefficiencies from a bounded-rationality perspective, providing robust theoretical foundations for healthcare policy incentives (14, 15). They contend that the healthcare market exhibits substantial non-rational decision-making leading to opting-out behaviors. These phenomena can be explained by theories such as reference dependence and framing effects (16).

**Figure 1:**
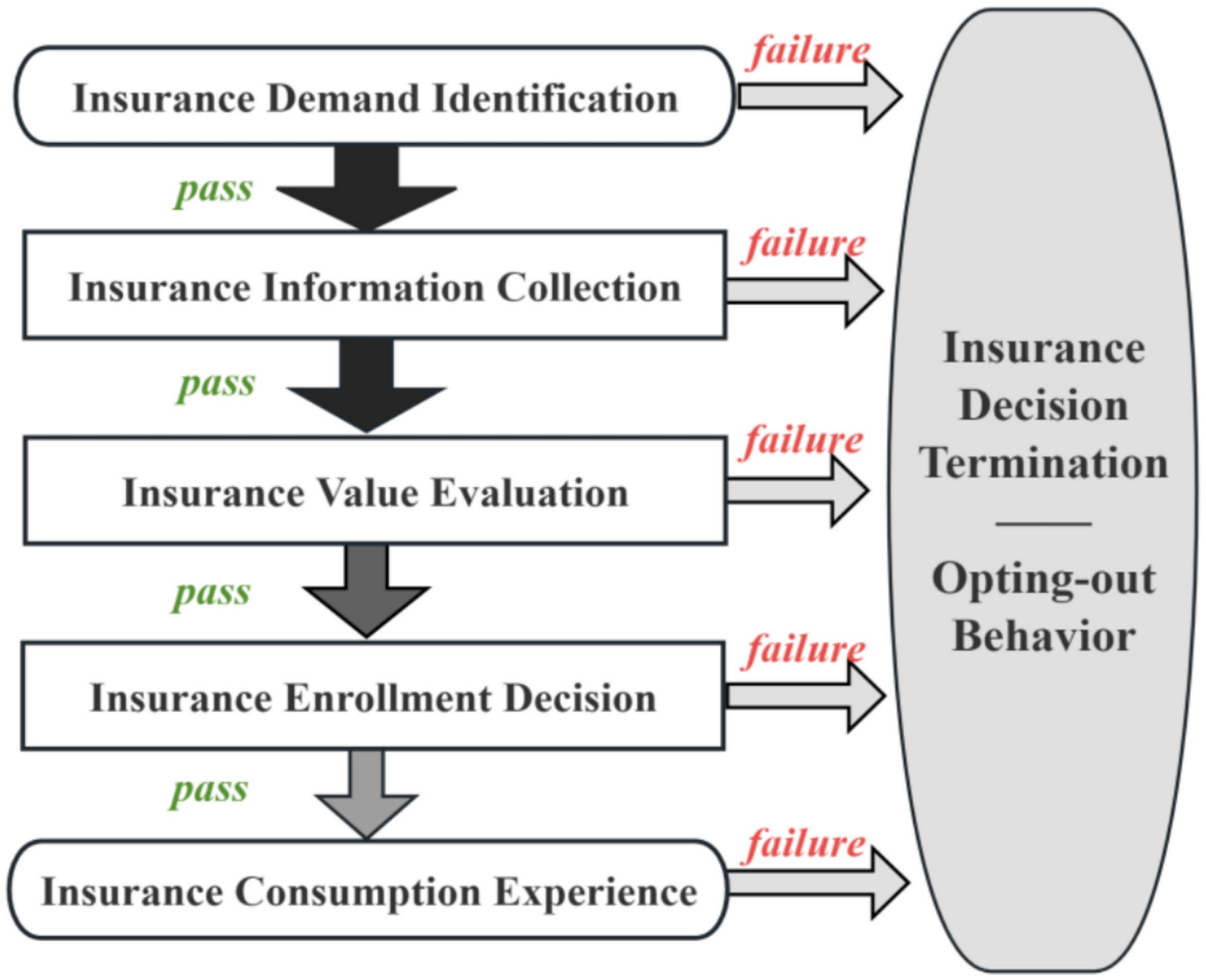
Schematic diagram of opting-out behavior generation logic.

Building on both adverse selection (rational choice under asymmetric information) and prospect theory (bounded rationality), we conceptualized basic medical insurance participation as a sequential consumer decision process (17). Specifically, drawing on consumption decision theory, we decompose the enrollment decision into five stages (18, 19): (i) recognizing the need for financial protection against health shocks, (ii) accessing and understanding enrollment rules and benefit information, (iii) evaluating expected value relative to premiums and opportunity costs, (iv) completing enrollment and payment given administrative and financial constraints, and (v) forming perceptions based on service access and reimbursement experience that shape renewal in the subsequent cycle. Opting out occurs when progression stalls at any stage due to structural barriers (e.g., portability and transaction costs) or behavioral frictions (e.g., limited insurance literacy, low perceived risk, and reference-dependent evaluations). Table 1 links these mechanisms to the survey-based proxies used in our empirical specification (see Figure 1).

**Table 1:** Mechanism-variable mapping across the enrollment decision process.

Decision stage (Figure 1)	Mechanisms	Variables (proxy)	Expected pattern (opt out)
Need recognition	Risk perception; insurance literacy; salience of illness	Age; education; marital status; recent health status	Higher: younger, lower education, unstable marital status, no recent illness
Information acquisition	Search and access costs; channel availability	Education; employment status; mobility range; expected local stay	Higher: lower education, precarious employment, higher mobility, short/uncertain stay
Value appraisal	Perceived value; trust; expected portability; framing/reference points	Mobility range; expected local stay; reason for migration; region	Higher: higher mobility, low settlement intention; lower: business migration
Enrollment and payment	Liquidity constraints; affordability; administrative burden	Household income; household expenditure; employment status; mobility range	Higher: lower income, higher budget constraints; lower: stable employment
Post-enrollment experience and renewal	Service access across regions; reimbursement frictions; perceived convenience	Mobility range; migration duration; expected local stay; region	Higher: higher mobility, weak local attachment

Listed variables are conceptual proxies and do not directly measure each mechanism.

It should be noted that due to limitations in data availability, this framework serves only as an explanatory guide for selecting variables and discussing results. Utilizing appropriate survey data as proxy variables and employing a logistic regression model to systematically explore the opting-out behavior of China’s migrant population is encouraged.

## 2 Methods

### 2.1 Data sources and sample selection

Migrant populations refer to individuals who engage in economic activities such as employment, business, or social services outside their registered residence without altering their household registration status. The core criterion is the separation of residence and household registration, excluding population movements due to tourism, education, visiting friends or relatives, or military service. The author obtained usage rights to the 2018 National Migrant Population Dynamic Monitoring Data from the National Health Commission, which includes 152,000 valid survey questionnaires from migrants.

As this study primarily focuses on participation in basic social medical insurance, 7,952 samples with “unclear” insurance enrollment status were excluded. The final analysis sample comprised 144,048 respondents who clearly stated their insurance status. Among them, 8,355 respondents indicated that they were not enrolled in any health insurance program at the time of the survey, representing an opt-out rate of 5.80%. This rate exceeds China’s current overall opt-out rate and warrants attention (11).

### 2.2 Descriptive analysis of opt-out rates by migrant characteristics

For the 144,048 migrant population samples, the following analysis compares opt-out rates across different migrant groups across five dimensions: demographics, migration patterns, economic status, employment and health.

#### 2.2.1 Opt-out rates among samples with different demographic characteristics

Demographic characteristics reflect the intrinsic status of the sample, including gender, age, marital status, and educational attainment. First, regarding gender, the opt-out rates for females and males in the migrant population sample were 6.14 and 5.48%, respectively. This indicates that female migrants have a significantly higher opt-out rate than males, making them more vulnerable to losing medical insurance coverage. Second, regarding age, the youngest individual who opted out of insurance coverage was 15 years old (the minimum age for survey participation was 15 years), while the oldest was 89 years. The average age was 35.79 years, with a variance of 11.78 years. Among these, the opt-out rates for the 15–19 and 20–24 age groups were relatively high at 10.86 and 7.5%. while other age groups hovered around 5.50%. Third, examining marital status reveals that newly married migrants exhibited the lowest opt-out rate (5.05%). Rates progressively increased for widowed (7.85%), unmarried (8.32%), remarried (8.56%), divorced (9.64%), and cohabiting (12.09%) individuals. This indicates that unstable marital status among the migrant population negatively impacts individuals’ willingness to participate in insurance to a certain extent. Finally, regarding educational attainment, the opt-out rate was higher among migrant samples with lower levels of education. The highest opt-out rate was observed among those who had not completed primary school (9.77%).

#### 2.2.2 Opt-out rates among samples with different migration characteristics

Migration characteristics reflect the mobility attributes of the sample, such as the migration scope, reasons for migration, migration duration, and migration plans. First, regarding the migration scope, the larger the migration range, the higher the opt-out rate. The opt-out rate for samples migrating across counties within the same city was only 3.45%, while it was 7.20% for samples migrating across provinces. Second, regarding migration reasons, samples migrating for employment or business purposes exhibited relatively lower opt-out rates of 5.71 and 4.99%, respectively. In contrast, migration due to family relocation or marriage resulted in higher opt-out rates of 7.45 and 7.35%. Third, regarding migration duration, the opt-out rates among samples with different migration periods showed slight variation, clustering closely around the mean of 5.80%. Only samples with migration durations exceeding 20 years exhibited a relatively higher opt-out rate. Finally, regarding the anticipated duration of local residence, migrant populations generally expressed uncertainty about long-term development plans, which dampened their enthusiasm for insurance participation. Notably, among those who did not intend to continue migrating, the opt-out rate was as high as 9.04%.

#### 2.2.3 Opt-out rates among samples with different economic characteristics

Economic characteristics reflect the household economic status of the sample, such as average monthly household income and expenditures. Regarding average monthly household income, the mean for the entire migrant population sample was 7,866.40 yuan, with a standard deviation of 8,247.45 yuan. Migrant samples with lower average monthly household income exhibited higher opt-out rates. Samples with zero or negative monthly household income had the highest opt-out rate (10.74%). On the other hand, regarding monthly household expenditure, the overall migrant population sample had a mean of 3,030.68 yuan and a standard deviation of 2,495.16 yuan. Among these, samples with monthly household expenditures below 1,500 yuan had an opt-out rate of 6.97%, which was significantly higher than that of samples in other expenditure brackets.

#### 2.2.4 Opt-out rates among samples with different employment characteristics

Employment characteristics reflect the employment status of the sample, such as employment status and working hours. Regarding employment status, employees with a fixed employer accounted for 45.20%, and their opt-out rate was the lowest at only 4.71%. Self-employed workers relying on skills or techniques had an intermediate opt-out rate. Meanwhile, workers without a fixed employer had an opt-out rate of 7.23%, approaching the 8.58% rate of the unemployed sample group. Additionally, regarding weekly working hours, the sample group working 29–42 h per week exhibited the lowest opt-out rate at 4.58%. Conversely, the sample group working 1–28 h per week recorded an opt-out rate of 7.17%. This indicates that samples with unstable employment and shorter working hours are more likely to opt out.

#### 2.2.5 Opt-out rates among samples with different health characteristics

Health characteristics reflect the health status and hygiene levels of the sample, such as the overall health status and recent physical condition. In China, the migrant population primarily originates from rural areas with high productive capacity, and their physical health levels are generally good. Among the sample, 86.8% self-reported being in good health, and 88.6% had no illness or injury in the past year. Their opt-out rates were close to the overall sample opt-out rate of 5.80%. Conversely, the opt-out rate among those who experienced illness requiring hospitalization within the past year was notably lower at 4.85%. This suggests that recent substantial medical expenditures may incentivize individuals to maintain insurance coverage, reflecting adverse selection within the health insurance system. However, it is noteworthy that the opt-out rate among samples who experienced illness but no hospitalization in the past year reached 6.66%, while those unable to care for themselves had an even higher opt-out rate of 11.63%. These individuals may have lost confidence in treatment due to long-term chronic health issues. Overall, if migrants face only short-term health risks, their attitudes toward insurance enrollment tend to be positive. However, if they endure prolonged poor health, their attitudes toward enrollment become increasingly negative.

### 2.3 Regression analysis of factors influencing opting-out behavior

The preceding analysis of opt-out rates among migrant samples provides an initial understanding of their current situation. However, to accurately identify the key factors influencing opting-out behavior, further regression analysis is necessary. Given that China’s basic medical insurance operates on a voluntary enrollment model, individual enrollment decisions are influenced by both macro-level policies and micro-level variables such as demographic characteristics, migration patterns, employment status, economic conditions, and health status (2, 20). However, considering that medical insurance policies constitute an exogenous institutional variable beyond individual control and are not the primary focus of this study, our empirical analysis will concentrate on examining the association between individual factors and opting-out decisions of the migrant population.

#### 2.3.1 Binary logistic regression model

Current domestic and international research on factors influencing health insurance participation typically employs discrete choice models, such as logistic and probit models, as well as Anderson’s behavioral models (21, 22). For this study, considering the objective realities of migrant populations enrolling in health insurance, the binary logistic regression model may be more suitable for our research needs. While our primary analysis uses logistic regression, a robustness check using a probit model yielded substantively identical conclusions. Future studies employing longitudinal data or multilevel modeling could further elucidate causal pathways and contextual effects. The specific regression model is as follows:


$$\ln[p/(1-p)]=\alpha +\beta_{1}x_{1}+\beta_{2}x_{2}+\cdots +\beta_{k}x_{k}$$ (1)

Applying Equation 1 to the analysis of migrant populations opting-out behavior, *p* represents the probability of opting-out behavior occurring, while (1 − *p*) denotes the probability of opting-out behavior not occurring. Thus, *p*/(1 − *p*) signifies the ratio of the probability of the event occurring to the probability of it not occurring, denoted as Odds Ratio (OR), with values ranging from 0 to positive infinity. α represents the constant term, *x_k_* denotes the various explanatory variables influencing the event occurrence, and *β_k_* represents the estimated coefficients of these explanatory variables. Finally, by interpreting *β_k_*, we determined the direction and magnitude of each explanatory variable’s impact on opting-out behavior. A positive *β_k_* (OR>1) indicates increased odds of opting out, a negative *β_k_* (OR<1) indicates decreased odds.

#### 2.3.2 Selection of explanatory variables

Based on the preceding analysis of the causes for opting out among migrant populations, the binary logistic regression model initially incorporated 14 explanatory variables across demographic, migration, employment, economic, and health domains. The sample’s geographic region was also set as a control variable.

Using SPSS 22.0, model diagnostics were conducted on the 144,048 migrant sample data. First, multicollinearity among the explanatory variables was tested. Linear regression was performed with opt-out status as the dependent variable and 15 explanatory variables as independent variables. According to multicollinearity diagnostics, the Variance Inflation Factor (VIF) for all 15 explanatory variables did not exceed 2. This indicates that while correlations exist among variables, they are not severe. Particularly given the large sample size, this level of multicollinearity was considered negligible. Next, the necessary model validity checks were performed. Analysis of the chi-square values for step size, block, and model revealed all significance levels below 0.05. This confirms a significant linear relationship between all explanatory variables and ln[p/(1−p)] , making model adoption a sound decision. Finally, based on the conceptual framework outlined in Table 1, we specified a binary logistic regression model including all hypothesized explanatory variables (demographic, migration, economic, employment, health) and the region control variable. The model was estimated using the enter method. The specific variables and their descriptions are presented in Table 2.

**Table 2:** Variables for analysis of factors influencing opting-out behavior.

Explanatory variable type	Variable	Variable description
Demographic characteristics variable	Age	15–19 years old: 1; 20–24 years old: 2; 25–29 years old: 3; 30–34 years old: 4; 35–39 years old: 5; 40–44 years old: 6; 45–49 years old: 7; 50–54 years old: 8; 55–59 years old: 9; 60 years old and above: 10
	Marital status	Unmarried: 1; first marriage: 2; remarried: 3; divorced: 4; widowed: 5; cohabiting: 6
	Educational attainment	No elementary education: 1; elementary education: 2; junior high education: 3; high school or vocational education: 4; college or technical education: 5; bachelor’s degree: 6; graduate degree: 7
Migration characteristics variable	Migration duration	Continuous variables (months)
	Mobility range	Inter-provincial: 1; inter-city within province: 2; inter-county within city: 3
	Reason for migration	Labor migration: 1; business activities: 2; accompany-migrated: 3; visiting relatives or birth: 4; marriage or moving, etc.: 5
	Expected local stay duration	No intention to stay locally: 1; undecided about staying locally: 2; will stay locally but undecided on duration: 3; will stay locally for under 4 years: 4; will stay locally for 5–9 years: 5; will stay locally for over 10 years: 6; will stay locally and settle permanently: 7
Economic Characteristics variable	Average monthly household income	Less than zero: 1; 0–4,000 yuan: 2; 4,000–8,000 yuan: 3; 8,000–12,000 yuan: 4; 12,000–16,000 yuan: 5; 16,000–20,000 yuan: 6; Over 20,000 yuan: 7
	Average monthly household expenditure	Less than 1,500 yuan: 1; 1,500–3,000 yuan: 2; 3,000–4,500 yuan: 3; 4,500–6,000 yuan: 4; Over 6,000 yuan: 5
Employment characteristics variable	Employment status	Unemployed: 1; workers without a fixed employer: 2; self-employed workers: 3; workers with a fixed employer: 4; employers: 5; other: 6
Health variables	Recent health status	Illness requiring hospitalization: 1; no illness: 2; illness without hospitalization: 3
Control variables	Current region	Eastern Region = 1; Central Region = 2; Western Region = 3; Northeast Region = 4

## 3 Results

### 3.1 Results of binary logistic regression analysis

Using a binary logistic regression model to analyze the factors influencing opting-out behavior, the results are shown in Table 3.

**Table 3:** Binary logistic regression analysis results for factors influencing opting out.

Explanatory variables	*B*	S. E.	Wald	Sig.	Exp (B)
1. Age group (ref: 15–19 years old)			161.506	0.000	
20–24 years old	−0.191***	0.065	8.595	0.003	0.826
25–29 years old	−0.278***	0.067	17.079	0.000	0.757
30–34 years old	−0.311***	0.071	19.233	0.000	0.733
35–39 years old	−0.328***	0.074	19.812	0.000	0.721
40–44 years old	−0.436***	0.076	32.776	0.000	0.647
45–49 years old	−0.431***	0.077	31.261	0.000	0.650
50–54 years old	−0.534***	0.083	41.755	0.000	0.586
55–59 years old	−0.751***	0.100	56.306	0.000	0.472
60 years and older	−1.052***	0.095	122.175	0.000	0.349
2. Gender (ref: male)	0.003	0.025	0.014	0.906	1.003
3. Educational attainment (ref: no elementary education)			155.304	0.000	
Elementary education	−0.412***	0.064	40.733	0.000	0.663
Junior high education	−0.668***	0.063	112.504	0.000	0.512
High school or vocational education	−0.600***	0.066	81.823	0.000	0.549
College or technical education	−0.633***	0.073	75.880	0.000	0.531
Bachelor’s degree	−0.825***	0.081	103.076	0.000	0.438
Graduate degree	−0.995***	0.191	27.053	0.000	0.370
4. Marital status (ref: first marriage)			342.397	0.000	
Unmarried	0.544***	0.041	174.491	0.000	1.723
Remarriage	0.499***	0.063	62.439	0.000	1.648
Divorced	0.654***	0.068	91.626	0.000	1.922
Widowed	0.302***	0.110	7.461	0.006	1.352
Cohabitation	0.744***	0.082	83.008	0.000	2.105
5. Mobility range (ref: interprovincial)			561.436	0.000	
Intra-provincial cross-city	−0.476***	0.028	291.117	0.000	0.621
Intra-city cross-county	−0.848***	0.041	435.227	0.000	0.428
6. Migration duration (continuous)	0.000**	0.000	6.544	0.011	1.000
7. Reasons for migration (ref: employment)			33.045	0.000	
Business	−0.224***	0.041	30.230	0.000	0.800
accompany-migrated	−0.057	0.042	1.785	0.181	0.945
Relatives/birth	0.044	0.092	0.225	0.635	1.045
Marriage/relocation, etc.	0.030	0.055	0.298	0.585	1.030
8. Expected local stay duration (ref: no intention to continue staying locally)			116.681	0.000	
Undecided about staying locally	−0.333***	0.072	21.185	0.000	0.717
Will stay locally but undecided on duration	−0.414***	0.070	34.941	0.000	0.661
Remaining locally for less than 4 years	−0.480***	0.073	43.018	0.000	0.619
Remaining locally for 5–9 years	−0.550***	0.079	47.959	0.000	0.577
Remaining locally for 10 years or more	−0.435***	0.077	31.789	0.000	0.648
Remaining in the local area, settling down	−0.201***	0.070	8.200	0.004	0.818
9. Average monthly household income (ref: negative average monthly income)			59.223	0.000	
0 < Monthly income ≤ 4,000 yuan	−0.238	0.185	1.645	0.200	0.788
4,000 yuan < monthly income ≤ 8,000 yuan	−0.367**	0.185	3.925	0.048	0.693
8,000 yuan < monthly income ≤ 12,000 yuan	−0.502***	0.187	7.209	0.007	0.605
12,000 yuan < monthly income ≤ 16,000 yuan	−0.558***	0.194	8.294	0.004	0.572
16,000 yuan < monthly income ≤ 20,000 yuan	−0.507**	0.199	6.459	0.011	0.602
20,000 yuan < average monthly income	−0.753***	0.205	13.425	0.000	0.471
10. Average monthly household expenditure (ref: average monthly expenditure ≤ 1500yuan)			16.651	0.002	
1,500 yuan < monthly expenditure ≤ 3,000 yuan	−0.093***	0.030	9.635	0.002	0.911
3,000 yuan < monthly expenditure ≤ 4,500 yuan	−0.102**	0.039	6.723	0.010	0.903
4,500 yuan < monthly expenditure ≤ 6,000 yuan	−0.087*	0.049	3.142	0.076	0.916
6,000 yuan < monthly expenditure	0.055	0.064	0.735	0.391	1.057
11. Employment identity (ref: unemployed)			398.306	0.000	
Workers without a fixed employer	−0.352***	0.066	28.584	0.000	0.704
Self-employed workers	−0.380***	0.063	36.415	0.000	0.684
Workers with a fixed employer	−0.838***	0.054	236.641	0.000	0.433
Employer	−0.165**	0.072	5.277	0.022	0.848
Other	−0.140	0.123	1.288	0.256	0.870
12. Weekly working hours (continuous)	0.001	0.001	1.587	0.208	1.001
13. Overall health level (ref: healthy)			4.581	0.205	
Basically healthy	0.017	0.038	0.191	0.662	1.017
Unhealthy but able to take care of oneself	−0.142*	0.082	2.962	0.085	0.868
Unable to take care of oneself	0.255	0.288	0.786	0.375	1.291
14. Health status in the past year (ref: illness requiring hospitalization)			19.477	0.000	
No illness	0.351***	0.080	19.476	0.000	1.421
Illness without hospitalization	0.275***	0.073	14.196	0.000	1.316
15. Region (ref: Eastern Region)			812.948	0.000	
Central Region	−0.280***	0.040	49.921	0.000	0.755
Western Region	−0.230***	0.030	58.462	0.000	0.795
Northeast Region	0.814***	0.038	451.539	0.000	2.256

***p < 0.01; **p < 0.05; *p < 0.1.

### 3.2 Interpretation of empirical findings

Among individual characteristic variables, three factors—age group, marital status, and educational attainment—significantly influenced the opt-out rate among migrants. First, compared to the 15–19 age group, migrants in all other age brackets exhibit lower tendencies to opt out. Moreover, the probability of opting out is negatively correlated with increasing age. Second, compared with migrants in first marriage, migrants in unmarried, remarried, divorced, widowed or cohabiting statuses all exhibited higher tendencies toward opting out. Finally, compared with migrants who did not accept elementary education, migrants with higher education levels showed lower tendencies to opt out. AS educational attainment increases, the tendency for migrant samples to opt out significantly decreases.

Among the mobility-related variables, three factors—mobility range, reasons for mobility, and planned local stay duration—significantly influenced the opt-out rate from medical insurance among migrants. First, compared to inter-provincial samples, both intra-provincial inter-city and intra-city inter-county samples exhibited a markedly reduced tendency to opt out of insurance. This indicates that greater geographical barriers faced by migrants are associated with more substantial objective obstacles to their participation in medical insurance. Second, compared with migrants moving for employment, those migrating for business purposes exhibit a significantly lower tendency to opt out of insurance. Migrants moving due to family relocation, birth, marriage, or moving house did not show significant differences in this regard. Third, migration duration shows a statistically significant but negligible association with the probability of choosing opt-out. Finally, regarding planned expected local stay duration, samples intending to remain and develop locally for longer periods exhibited lower opting-out tendencies than those not planning to stay.

Among economic characteristics, monthly household income and monthly household expenditure significantly influenced the opt-out rate among migrants. Although both variables assess household economic conditions, their mechanisms of influence on opting-out behavior differ. Monthly household income primarily reflects the overall family income. Compared to samples with household monthly income below zero, those with income between 0 and 4,000 yuan showed no significant difference, while samples with income above 4,000 yuan exhibited a lower tendency to opt out of insurance. Moreover, the probability of opting out decreases as income increases. In contrast, household monthly expenditure primarily reflects the overall expenditure pressure on the household. Compared to migrant samples with monthly household expenditures less than or equal to 1,500 yuan, those with higher monthly expenditures exhibited a lower tendency to opt out of insurance. However, samples with monthly expenditures exceeding 6,000 yuan did not show a reduced tendency to opt out of insurance. This may be because significant expenditure pressure is associated with reduced ability to pay premiums.

Among employment characteristic variables, employment status significantly influenced the opt-out rate among migrants. Compared with unemployed individuals, workers without a fixed employer, self-employed workers, and those with a fixed employer exhibited lower opting-out tendencies. Among these, workers with fixed employers had the lowest probability of opting out. Notably, the opting-out probability among the employer group—active participants in socioeconomic activities—showed no significant difference compared to the unemployed group.

Among health-related variables, the recent physical condition variable significantly influenced the opt-out rate among migrant populations. Compared to samples of migrants who were ill and hospitalized, those who were ill but not hospitalized and those who were not ill, both exhibited higher odds of opting out of insurance. Some healthy migrants believe that being young and strong makes participating in medical insurance meaningless and that it would instead waste premium expenses, so they choose to opt out of insurance. This indicates that adverse selection may exist in migrants’ insurance participation decisions.

## 4 Discussion

### 4.1 Key influencing factors of insurance opting-out behavior among migrants

Based on the preceding analysis of opt-out rates among migrants and the regression analysis of influencing factors, the significant impact of individual characteristics on opting-out behavior can be summarized in the following five aspects.

First, migrants who are younger, less educated, and in unstable marital relationships exhibit a significantly higher tendency to opt out of insurance. This is consistent with the view that opting-out behavior is an external manifestation of subjective judgment, closely tied to an individual’s basic literacy in healthcare security (23, 24). Younger individuals have less experience with health risks and correspondingly feel less pressure to maintain coverage. Lower educational attainment is associated with limited ability to access and comprehend health insurance information (25, 26). Meanwhile, unstable marital status is correlated with lower motivation and capacity to pursue health security.

Second, migrant populations with higher mobility and broader migration ranges exhibit significantly higher tendencies to opt out of insurance. Owing to regional fragmentation in healthcare systems, migrants who frequently move between different areas struggle to fully utilize their insurance benefits. Higher costs associated with insurance utilization are correlated with a stronger motivation to opt out (27, 28).

Third, migrants with lower average monthly household income and expenditure exhibit a significantly higher tendency to opt out of insurance. Lower household income is associated with reduced financial capacity to cover premiums for the individual (29, 30). For samples with average household income below 4,000 yuan, opting-out behavior is often more of a helpless move (25). Additionally, household expenditures exceeding normal levels may not indicate affluence but rather may be linked to lower enrollment.

Fourth, migrants with less stable employment and inconsistent employers exhibit a significantly higher tendency to opt out of insurance. Stable employment and employers imply a higher probability of participating in employee medical reimbursement programs (31). Moreover, sustained income security is linked to higher development expectations and greater eagerness for health coverage among migrants (32).

Fifth, healthier migrants exhibit a significantly higher tendency to opt out of insurance. However, within the adverse selection dynamics of this population, only short-term health risks and substantial medical expenses reduce the opt-out rate (33–35). Patients with chronic diseases and poor prognosis did not exhibit a lower opt-out rate.

### 4.2 Health insurance policy implications for reducing opt-out rates among migrants

To reduce opt-out rates of insurance among migrants and better achieve UHC, this study suggests that health insurance policies can provide targeted support across the five stages of enrollment decision-making.

During the needs identification phase, intensify enrollment outreach and guidance for younger, less educated migrants (36, 37). Health insurance authorities should enhance their understanding of these vulnerable groups and collaborate with other social forces to implement more accessible enrollment guidance initiatives. Use clear, accurate data to describe the incidence rates and treatment costs of common diseases, while employing vivid, relatable case studies to convey the value of health insurance (11, 24, 38).

In the information collection phase, improve the communication system for health insurance information to ensure migrants’ access to channels (11, 39). To guarantee information availability in both their registered hometowns and work locations, health insurance departments could consider assigning one volunteer per community or village to promote health insurance policies. Trained volunteers can facilitate face-to-face offline communication, ensuring effective information dissemination to all individuals in the area.

In the insurance value evaluation phase, the convenience of cross-regional medical treatment is enhanced, and the cost of medical insurance usage for migrants is reduced, which could strengthen their motivation to participate in insurance programs (25, 27). Additionally, to mitigate the impact of adverse selection effects, health insurance departments need to comprehensively identify and publicize the potential disease risks faced by healthy migrants (40). This is particularly crucial for flexible workers who are not covered by employee medical insurance, as it is essential to make them aware of the health threats associated with their current occupational activities (41, 42).

Fourth, in the enrollment decision phase, to enhance affordability for migrant populations, health insurance departments should offer more diverse enrollment options while identifying genuinely economically disadvantaged groups to provide the necessary premium support (43). Concurrently, these departments should strengthen interregional coordination to lower barriers and operational difficulties for cross-regional enrollment among migrant populations (44).

Fifth, in the area of insurance consumption experience, streamline the reimbursement process for medical treatment received outside the insured’s registered locality while enhancing the level of medical insurance benefits for cross-regional claims (25, 27, 45). Medical insurance authorities must promptly expand the scope of coordinated coverage to ensure adequate protection for migrants seeking medical care outside their home country (39, 46). Only by continuously improving the experience and recognition of medical insurance among this mobile population can we sustainably increase coverage rates and achieve universal health coverage.

### 4.3 Strengths and limitations

This study applies consumer decision theory to analyze opting out of insurance among migrants, decomposing the enrollment decision process into five steps to construct the fundamental logic behind opting-out behavior. Using a nationwide sample of China’s migrant population for empirical analysis, it explores key factors influencing opting-out and proposes targeted policy recommendations.

It should be noted that the empirical data for this study originates from 2018, presenting some limitations in timeliness. However, considering that China’s medical insurance policies have not undergone significant adjustments since 2018, maintaining consistent fundamental structures and operational logic with only developmental variations, the analytical conclusions of this study should retain practical relevance. In addition, the lack of sampling weights for the samples is also a limitation of this study. Future research should utilize restricted access data with complete sampling parameters and verify these findings through weighted analysis.

## 5 Conclusion

This study examines health insurance opting-out behavior among China’s migrant population, mapping and comparing opt-out rates across different demographic segments to reveal how individual factors influence this behavior. Findings indicate that younger age, lower education levels, unstable marital status, higher mobility, broader migration scope, tighter household finances, less stable employment, and better physical health are significantly associated with increased opting-out tendencies. To reduce opt-out rates, medical insurance authorities should supplement enrollment policies with targeted support, assisting more migrants in navigating the five decision-making steps for enrollment to secure healthcare coverage and protection.

## Data Availability

Publicly available datasets were analyzed in this study. This data can be found at: https://www.ncmi.cn/phda/dataDetails.do?id=CSTR:17970.11.A000T.202205.84.V1.0.
