# Supplementary material for: Factors influencing the decision to opt out of basic medical insurance among China’s migrant population: a logistic regression analysis
Source: Front Public Health. 2026 Mar 24;14:1787324. doi: 10.3389/fpubh.2026.1787324 (PMC13053514; doi:10.3389/fpubh.2026.1787324)
Supplement: Supplementary file 1 [file Supplementary_file_1.pdf]

## Appendix 1

Table 1. Chi-square test of key variables between included and excluded samples

| Variable | Test statistic | P value | Effect size |
|---|---|---|---|
| Gender | $\chi^2(1)$ =2.541 | 0.111 | Phi = .004 |
| Age | $\chi^2(9)$ = 1220.625 | <0.001 | Cramer's V = 0.090 |
| Marital Status | $\chi^2(5)$ = 1497.794 | <0.001 | Cramer's V = 0.099 |
| Educational Attainment | $\chi^2(6)$ = 345.646 | <0.001 | Cramer's V = 0.048 |
| Mobility Range | $\chi^2(2)$ = 419.100 | <0.001 | Cramer's V = 0.053 |
| Reason for Migration | $\chi^2(8)$ = 406.778 | <0.001 | Cramer's V = 0.052 |
| Overall health level | $\chi^2(3)$ = 7.462 | 0.059 | Cramer's V = 0.007 |
| Average Monthly Household Expenditure | $\chi^2(4)$ = 132.262 | <0.001 | Cramer's V = 0.029 |
| Employment Status | $\chi^2(5)$ = 672.057 | <0.001 | Cramer's V = 0.066 |
| Current Region | $\chi^2(3)$ = 601.205 | <0.001 | Cramer's V = 0.063 |

## Appendix 2

Table 2. The comparative analysis of Probit regression results and logistic regression results

| **Explanatory Variables** | **B (logistic)** | **B (probit)** | **Consistent direction** | **consistent significance** | **proportion （logistic/ /probit）** |
|---|---|---|---|---|---|
| 1. Age Group (Ref: 15-19 years old) | | | | | |
| 20-24 years old | -0.191*** | -0.107*** | √ | √ | 1.79 |
| 25-29 years old | -0.278*** | -0.149*** | √ | √ | 1.87 |
| 30-34 years old | -0.311*** | -0.165*** | √ | √ | 1.88 |
| 35-39 years old | -0.328*** | -0.171*** | √ | √ | 1.92 |
| 40-44 years old | -0.436*** | -0.219*** | √ | √ | 1.99 |
| 45-49 years old | -0.431*** | -0.221*** | √ | √ | 1.95 |
| 50-54 years old | -0.534*** | -0.264*** | √ | √ | 2.02 |
| 55-59 years old | -0.751*** | -0.362*** | √ | √ | 2.07 |
| 60 years and older | -1.052*** | -0.510*** | √ | √ | 2.06 |
| 2. Gender (Ref: Male) | 0.003 | -0.003 | × | √ | 1.00 |
| 3. Educational Attainment (Ref: No elementary education ) | | | | | |

| | | | | | |
|---|---|---|---|---|---|
| Elementary education | -0.412*** | -0.200*** | √ | √ | 2.06 |
| Junior high education | -0.668*** | -0.323*** | √ | √ | 2.07 |
| High school or vocational education | -0.600*** | -0.290*** | √ | √ | 2.07 |
| College or technical education | -0.633*** | -0.309*** | √ | √ | 2.05 |
| Bachelor's degree | -0.825*** | -0407*** | √ | √ | 0.00 |
| Graduate degree | -0.995*** | -0.495*** | √ | √ | 2.01 |
| 4. Marital Status (Ref: First Marriage) | | | | | |
| Unmarried | 0.544*** | 0.258*** | √ | √ | 2.11 |
| Remarriage | 0.499*** | 0.241*** | √ | √ | 2.07 |
| Divorced | 0.654*** | 0.323*** | √ | √ | 2.02 |
| Widowed | 0.302*** | 0.149*** | √ | √ | 2.03 |
| Cohabitation | 0.744*** | 0.373*** | √ | √ | 1.99 |
| 5. Mobility Range (Ref: Interprovincial) | | | | | |
| Intra-provincial cross-city | -0.476*** | -0.226*** | √ | √ | 2.11 |
| Intra-city cross-county | -0.848*** | -0.396*** | √ | √ | 2.14 |
| 6. Migration Duration (Continuous) | 0.000** | 0.000** | √ | √ | 1.00 |
| 7. Reasons for Migration (Ref: Employment) | | | | | |
| Business | -0.224*** | -0.107*** | √ | √ | 2.09 |
| accompany-migrated | -0.057 | -0.029 | √ | √ | 1.97 |
| Relatives/Birth | 0.044 | 0.030 | √ | √ | 1.47 |
| Marriage/Relocation etc. | 0.030 | 0.012 | √ | √ | 2.50 |
| 8. Expected Local Stay Duration (Ref: No intention to continue staying locally) | | | | | |
| Undecided about staying locally | -0.333*** | -0.165*** | √ | √ | 2.02 |
| Will stay locally but undecided on duration | -0.414*** | -0.206*** | √ | √ | 2.01 |
| Remaining locally for less than 4 years | -0.480*** | -0.239*** | √ | √ | 2.01 |
| Remaining locally for 5-9 years | -0.550*** | -0.264*** | √ | √ | 2.08 |
| Remaining locally for 10 years or more | -0.435*** | -0.215*** | √ | √ | 2.02 |
| Remaining in the local area, settling down | -0.201*** | -0.105*** | √ | √ | 1.91 |
| 9. Average Monthly Household Income (Ref: Negative Average Monthly Income) | | | | | |
| 0 < Monthly Income≤4000 yuan | -0.238 | -0.122 | √ | √ | 1.95 |
| 4,000 yuan < monthly income≤8,000 yuan | -0.367** | -0.185* | √ | √ | 1.98 |
| 8,000 yuan < monthly income≤12,000 yuan | -0.502*** | -0.247** | √ | √ | 2.03 |
| 12,000 yuan< monthly income≤16,000 yuan | -0.558*** | -0.276*** | √ | √ | 2.02 |
| 16,000 yuan< monthly income≤20,000 yuan | -0.507** | -0.253** | √ | √ | 2.00 |
| 20,000 yuan < average monthly income | -0.753*** | -0.364*** | √ | √ | 2.07 |
| 10. Average monthly household expenditure (ref: average monthly expenditure⩽1500yuan) | | | | | |
| 1,500 yuan< monthly expenditure⩽3000 yuan | -0.093*** | -0.044*** | √ | √ | 2.11 |
| 3,000 yuan< monthly expenditure⩽4500 yuan | -0.102** | -0.048** | √ | √ | 2.13 |
| 4,500 yuan<monthly expenditure⩽6,000 yuan | -0.087* | -0.041* | √ | √ | 2.12 |
| 6,000 yuan < monthly expenditure | 0.055 | 0.026 | √ | √ | 2.12 |
| 11. Employment Identity (Ref: Unemployed) | | | | | |
| Workers without a fixed employer | -0.352*** | -0.171*** | √ | √ | 2.06 |

| | | | | | |
|---|---|---|---|---|---|
| Self-employed workers | -0.380*** | -0.186*** | √ | √ | 2.04 |
| Workers with a fixed employer | -0.838*** | -0.400*** | √ | √ | 2.10 |
| Employer | -0.165** | -0.079** | √ | √ | 2.09 |
| Other | -0.140 | -0.057 | √ | √ | 2.46 |
| 12. Weekly working hours (Continuous) | 0.001 | 0.000 | √ | √ | 1.00 |
| 13. Overall health level (Ref: Healthy) | | | | | |
| Basically healthy | 0.017 | 0.011 | √ | √ | 1.55 |
| Unhealthy but able to take care of oneself | -0.142* | -0.073* | √ | √ | 1.95 |
| Unable to take care of oneself | 0.255 | 0.126 | √ | √ | 2.02 |
| 14. Health status in the past year (Ref: Illness requiring hospitalization) | | | | | |
| No illness | 0.351*** | 0.166*** | √ | √ | 2.11 |
| Illness without hospitalization | 0.275*** | 0.129*** | √ | √ | 2.13 |
| 15. Region (Ref: Eastern Region) | | | | | |
| Central Region | -0.280*** | -0.122*** | √ | √ | 2.30 |
| Western Region | -0.230*** | -0.105*** | √ | √ | 2.19 |
| Northeast Region | 0.814*** | 0.414*** | √ | √ | 1.97 |
